# Hybrid Bright-Dark-Field Microscopic Fringe Projection System for Cu Pillar Height Measurement in Wafer-Level Package

**DOI:** 10.3390/s24165157

**Published:** 2024-08-09

**Authors:** Dezhao Wang, Weihu Zhou, Zili Zhang, Fanchang Meng

**Affiliations:** 1 College of Opto-Electronic Engineering, Changchun University of Science and Technology, Changchun 130022, China; wangdezhao@ime.ac.cn; 2 Photoelectric Technology R&D Center, Institute of Microelectronics of the Chinese Academy of Sciences, Beijing 100029, China; mengfanchang@ime.ac.cn; 3 University of Chinese Academy of Sciences, Beijing 100029, China

**Keywords:** microscopic fringe projection profilometry, three-dimensional sensing, semiconductor metrology, optical profilometer

## Abstract

Cu pillars serve as interconnecting structures for 3D chip stacking in heterogeneous integration, whose height uniformity directly impacts chip yield. Compared to typical methods such as white-light interferometry and confocal microscopy for measuring Cu pillars, microscopic fringe projection profilometry (MFPP) offers obvious advantages in throughput, which has great application value in on-line bump height measurement in wafer-level packages. However, Cu pillars with large curvature and smooth surfaces pose challenges for signal detection. To enable the MFPP system to measure both the top region of the Cu pillar and the substrate, which are necessary for bump height measurement, we utilized rigorous surface scattering theory to solve the bidirectional reflective distribution function of the Cu pillar surface. Subsequently, leveraging the scattering distribution properties, we propose a hybrid bright-dark-field MFPP system concept capable of detecting weakly scattered signals from the top of the Cu pillar and reflected signals from the substrate. Experimental results demonstrate that the proposed MFPP system can measure the height of Cu pillars with an effective field of view of 15.2 mm × 8.9 mm and a maximum measurement error of less than 0.65 μm.

## 1. Introduction

Cu pillars are physical interconnect structures utilized in wafer-level 3D chip stacking. The uniform height of copper pillars ensures chip yield [1], necessitating measurement of all Cu pillars on wafers in the semiconductor industry [2,3]. Traditional optical sensing techniques such as white-light interferometry [4,5,6], confocal microscopy [7,8], and white-light triangulation [9,10] are used in Cu pillar measurement. However, their inherent limitations in measurement speed pose challenges in achieving the required throughput for wafer inspection and metrology in industry, especially in on-line measurement. Hence, exploring alternative optical measurement techniques with superior throughput is essential.

Microscopic fringe projection profilometry (MFPP) [11] utilizes phase shifting profilometry (PSP) for 3D sensing. The wide field of view (FOV) of MFPP makes it potentially competitive in measurement throughput compared to other optical profilometry methods using microscopic objectives. While MFPP has successfully measured electronic packages [12,13,14] and solder bumps with a planar structure [15], it still faces challenges when applied to wafer-level structures [16]. In other words, the existing MFPP system cannot be directly used to measure Cu pillars because Cu pillars have micron-scale physical structures and surfaces with large curvature and extreme smoothness, making the MFPP system difficult for capturing enough optical signals for 3D reconstruction. Representative studies addressing the high dynamic range (HDR) issue are challenging to apply to Cu pillar measurements due to the rapid degradation of scattered light intensity caused by the non-coaxial illumination required for MFPP. Even with multiple exposures [17] or adjustments in illumination intensity [18], the scattered light intensity on the Cu pillar’s surface remains too weak for detection by state-of-the-art CMOS. As a result, the optical signal detection capability of the MFPP system’s imaging module does not match with the scattered light distribution, preventing the CMOS from covering the dynamic range of the scattered structured light. To the best of our knowledge, studies investigating the relationship between the scattering mechanisms of the surface under test and the detectable profile region of the MFPP system are lacking. Hence, this study will focus on the relationship between the intensity distribution of scattered light from the Cu pillar surface and the detectability of the MFPP system.

This study first analyzes the issue of detecting scattering signals caused by the surface characteristics of Cu pillars. Secondly, the concept of a hybrid bright-dark-field MFPP system is introduced, followed by an analysis of the relationship between scattered light detection capability and system geometry using the rigorous Rayleigh–Rice vector perturbation surface scattering theory. Subsequently, validity and performance verification experiments are conducted, applying the phase-shift algorithm and point cloud registration to the proposed MFPP system.

## 2. Issues with Large Curvature, Smooth Surface-Induced Light Signal Detection

**Figure 1:**
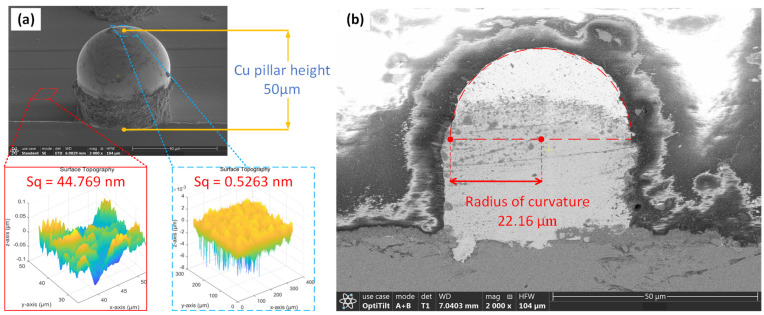
Characterization of the Cu pillar. (**a**) Roughness of the Cu pillar top and substrate. (**b**) Radius of curvature of the Cu pillar.

**Figure 2:**
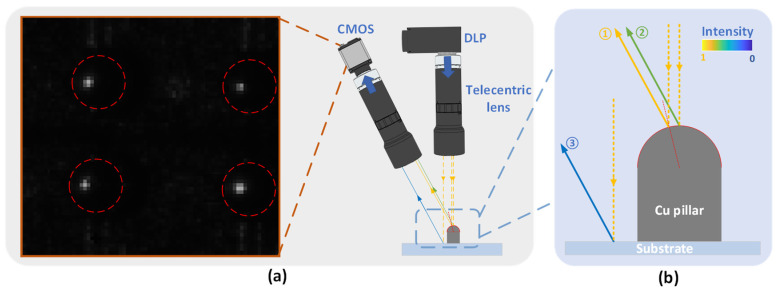
Images of the Cu pillar using the MFPP architecture with vertical projection and oblique imaging. (**a**) Highlighted areas are located on the sidewalls of the Cu pillar, with no light signals observed on the substrate or the top of the Cu pillar. The red circle represents the expected area of the Cu pillar. (**b**) The relative intensity of the beams entering the imaging module shows that scattered light intensity is significantly smaller than mirror range light intensity.

Surface characterization and profile analysis of the Cu pillar are required before analyzing the scattered light signal. Figure 1a shows a scanning electron microscope (SEM) image of the Cu pillar. The Cu pillar is hemispherical, 50 μm in height, and composed mainly of reflowed Sn-Ag solder. The substrate under the Cu pillar is a redistribution layer made of metallization material. Both materials are smooth, but their roughness varies significantly. The profile and roughness of both the Cu pillar and the substrate were measured using a white-light interferometer. Figure 1a shows roughness values of 0.52 nm for the Sn-Ag material atop the Cu pillar and 44.76 nm for the substrate. Figure 2 presents the exact cross-section image of the Cu pillar obtained through cross-section polishing. The Cu pillar is confirmed to be essentially spherical, with a radius of curvature of 22.16 μm.

In practice, the imaging systems face a signal acquisition challenge when surfaces under test have large curvature and smoothness due to a limited numerical aperture (NA). Unfortunately, MFPP systems using high-resolution telecentric lenses (typical NA = 0.05, f/number = 6) encounter greater difficulty in optical signal acquisition due to the smaller NA. Two typical MFPP system architectures include: (1) vertical illumination with oblique imaging [14] (or vice versa [13]), and (2) oblique illumination with oblique imaging [12]. While effective in conventional measurements, these architectures struggle to image both the top and substrate regions of a Cu pillar.

**Figure 3:**
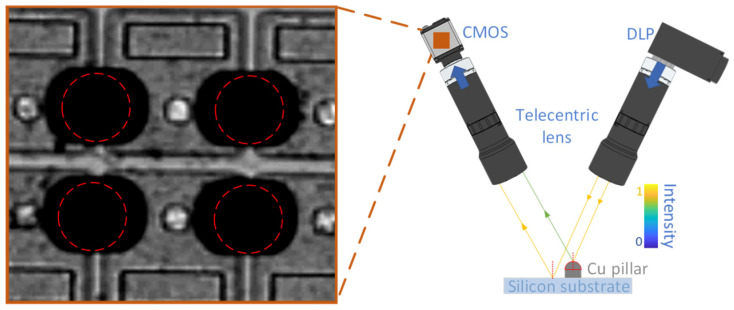
Images of the Cu pillar using the MFPP architecture with oblique projection and imaging. The substrate region alone can be imaged because of the significant roughness difference between the Cu pillar and the substrate. The red circle represents the expected area of the Cu pillar.

Figure 2a shows an image of the Cu pillar captured by the MFPP system using vertical projection and oblique imaging architecture. Except for the highlights on the sidewall of the Cu pillar, the rest of the image has insufficient light to form a meaningful image. Figure 2b qualitatively presents the relative intensity of specific beams. Vertical illumination results in beam-1 having the strongest intensity due to specular reflection, while beam-2 and beam-3 exhibit significantly lower intensity because smooth surfaces cause rapid light attenuation with an increased scattering angle. Notably, beam-2 has greater intensity than beam-3 due to surface roughness differences, despite sharing the same scattering angle. Due to intensity differences beyond the CMOS dynamic range, only beam-2 can be responded to, as shown in Figure 2a. Obviously, the height of the Cu pillar cannot be determined when the sinusoidal fringe pattern is projected. Similarly, by altering the system to an oblique projection and tilted imaging architecture, only the substrate regions that satisfy the specular reflection condition can be imaged. Likewise, changing the system to use oblique projection and imaging architecture allows imaging only of substrate regions that meet the specular reflection condition, as shown in Figure 3.

In conclusion, detecting weak signals poses the primary challenge for measuring the Cu pillar. In contrast to conventional HDR issues that deal with mitigating image saturation, measuring Cu pillars focuses on detecting dark areas due to weak scattering. In practice, even maximum illumination intensity fails to image dark regions due to their distinct generation mechanism from occlusion. Thus, to measure the height of the Cu pillar, the MFPP system needs to capture images of both the substrate and the dark area on top of the Cu pillar.

## 3. Methods

### 3.1. Concept of Hybrid Bright-Dark-Field MFPP System

#### 3.1.1. Optical System Architecture

**Figure 4:**
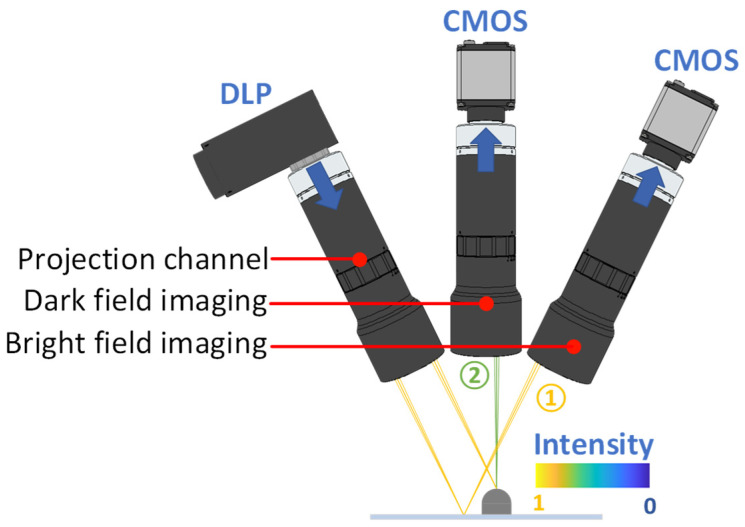
Concept of hybrid bright-dark-field MFPP system. Beam−2 represents the scatter light and beam−1 represents the reflected light from the substrate.

Dark-field scattering imaging has been extensively studied in microscopy and has successfully achieved 3D imaging for small structures [19,20,21]. Dark-field imaging effectively captures weakly scattered light signals from a structure with large curvature. Drawing inspiration from dark-field confocal microscopy, we introduce the dark-field imaging strategy into the MFPP system. Figure 4 illustrates the concept of a hybrid bright-dark-field MFPP system. As shown in Figure 4, with the oblique projection condition, the bright-field imaging channel captures specular reflection signals from the substrate, and the dark-field channel captures scattering signals from the top region of the Cu pillar. Ideally, maintaining constant illumination intensity while independently adjusting exposure times in the two imaging channels allows for sufficient light signal capture from both the top region of the Cu pillar (beam-2 in Figure 4) and the substrate (beam-1 in Figure 4). Subsequently, the height of the Cu pillar can be extracted by fusing the 3D point clouds from the two imaging channels.

The primary challenge for the proposed MFPP system concept is to maintain adequate modulation of scattered fringe patterns entering the dark-field imaging channel. The intensity of scattered light depends on both the illumination angle of incidence and the characteristics of the surface under test. Naturally, designing a viable MFPP system requires accurate prediction of the scattered light distribution from the Cu pillar.

#### 3.1.2. Spatial Distribution of Scattered Light on the Cu Pillar

To determine the detectable region of the MFPP system for the top region of the Cu pillar, we need to quantify the distribution of the scattered light field. We use the rigorous Rayleigh–Rice vector perturbative surface scattering theory to predict the scattering distribution, as the roughness shown in Figure 1 falls within the theory’s applicable range [22].

**Figure 5:**
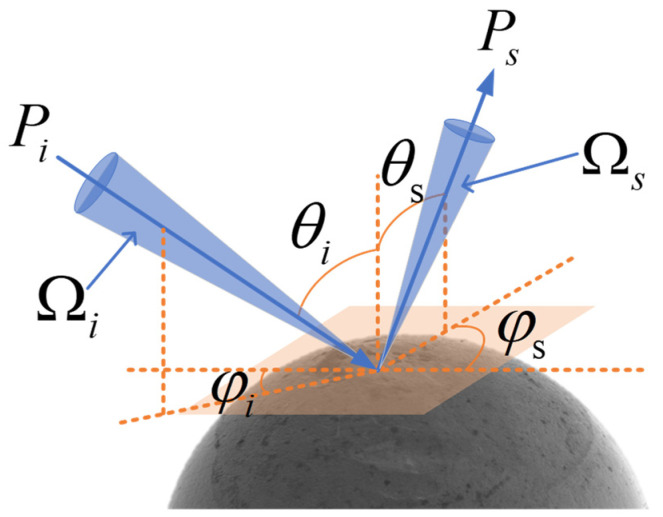
Definition of the BRDF in the spherical coordinate system.

First, the intensity distribution of the scattered light field is described by the bidirectional reflective distribution function (BRDF). As shown in Figure 5, the BRDF is related to $\theta_{i}$, $\varphi_{i}$, $\theta_{s}$, and $\varphi_{s}$. In the spherical coordinate system, the unit solid angle is Ω, θ and φ denote the polar angle and azimuthal angle, respectively, and the subscripts *i* and *s* denote the incident and scattered light, respectively. The BRDF is defined as the ratio of the radiance of the scattered light emitted from the unit solid angle at an arbitrary spatial angle ${{dL}_{s}(\theta }_{s},\varphi_{s})$, to the irradiance received per unit area at an arbitrary spatial angle ${dE}_{i}{(\theta }_{i},\varphi_{i})$:

(1) $$BRDF\left(\theta_{i},\varphi_{i},\theta_{s},\varphi_{s}\right)\equiv \frac{dL_{s}\left(\theta_{s},\phi_{s}\right)}{dE_{i}\left(\theta_{i},\phi_{i}\right)}=\frac{\frac{dP_{s}}{d\Omega_{s}}}{P_{i}\cos\theta_{s}}≅\frac{\frac{P_{s}}{\Omega_{s}}}{P_{i}\cos\theta_{s}}$$

where $P_{s}$ represents the power of scattered light, $P_{i}$ represents the power of incident light, and the unit of BRDF is $S_{r}^{-1}$.

The Rayleigh–Rice scattering theory relates the surface roughness to the scattered light field distribution; the *BRDF* is given by

(2) $$BRDF=\frac{\frac{dP}{d\Omega_{s}}}{P_{i}\cos\theta_{s}}=\left(\frac{16\pi^{2}}{\lambda^{4}}\right)\cos\theta_{i}\cos\theta_{s}\cdot Q\cdot \mathrm{PSD}(f_{x},f_{y})$$

where ${PSD(f}_{x},f_{y})$ represents the roughness power spectral density (*PSD*) of the surface under test, while $f_{x}$ and $f_{y}$ denote the spatial frequency of the profile in the *x* and *y* directions, respectively:

(3) $$\begin{array}{cc} f_{x}=\frac{\sin\theta_{s}\cos\phi_{s}}{\lambda }-\frac{\sin\theta_{i}}{\lambda }, & f_{y}=\frac{\sin\theta_{s}\sin\phi_{s}}{\lambda } \end{array}$$

where *Q* is the polarization factor, dependent on the material’s dielectric constant, angle of incidence, and scattering angle. Furthermore, *Q* relates to the polarization states of incident and scattered light. For unpolarized light, *Q* is given by

(4) $$\begin{array}{c} Q=\frac{1}{2}(Q_{ps}+Q_{pp}+Q_{sp}+Q_{ss})\\ Q_{ss}={\left|\frac{\left(\varepsilon -1)\cos\phi_{s}\right.}{\left(\cos\theta_{i}+\sqrt{\varepsilon -\sin^{2}\theta_{i}}\right)\left(\cos\theta_{s}+\sqrt{\varepsilon -\sin^{2}\theta_{s}}\right)}\right|}^{2}\\ Q_{sp}={\left|\frac{\left(\varepsilon -1)\sqrt{\varepsilon -\sin^{2}\theta_{s}}\sin\phi_{s}\right.}{\left(\cos\theta_{i}+\sqrt{\varepsilon -\sin^{2}\theta_{i}}\right)\left(\varepsilon \cos\theta_{s}+\sqrt{\varepsilon -\sin^{2}\theta_{s}}\right)}\right|}^{2}\\ Q_{ps}={\left|\frac{\left(\varepsilon -1)\sqrt{\varepsilon -\sin^{2}\theta_{i}}\sin\phi_{s}\right.}{\left(\varepsilon \cos\theta_{i}+\sqrt{\varepsilon -\sin^{2}\theta_{i}}\right)\left(\cos\theta_{s}+\sqrt{\varepsilon -\sin^{2}\theta_{s}}\right)}\right|}^{2}\\ Q_{pp}=\left|\frac{\left(\varepsilon -1)\left(\sqrt{\varepsilon -\sin^{2}\theta_{s}}\sqrt{\varepsilon -\sin^{2}\theta_{i}}\cos\phi_{s}-\varepsilon \sin\theta_{i}\cos\theta_{s}\right)\right.}{\left(\varepsilon \cos\theta_{i}+\sqrt{\varepsilon -\sin^{2}\theta_{i}}\right)\left(\varepsilon \cos\theta_{s}+\sqrt{\varepsilon -\sin^{2}\theta_{s}}\right)}\right| \end{array}$$

Since the material of the Cu pillar is a strongly absorbing medium with high reflectivity, the absolute value of its dielectric constant is much larger than $sin\theta_{i}$. Therefore, *Q* can be approximated as [23]:

(5) $$\begin{array}{c} Q=\frac{1}{2}(Q_{ps}+Q_{pp}+Q_{sp}+Q_{ss})\\ Q_{ss}=\cos^{2}\phi_{s}\\ Q_{sp}={\left(\frac{\sin\phi_{s}}{\cos\theta_{s}}\right)}^{2}\\ Q_{ps}={\left(\frac{\sin\phi_{s}}{\cos\theta_{i}}\right)}^{2}\\ Q_{pp}={\left[\frac{\left(\cos\phi_{s}-\sin\theta_{i}\sin\theta_{s}\right)}{\left(\cos\theta_{i}\cos\theta_{s}\right)}\right]}^{2} \end{array}$$

According to Equation (2), solving the BRDF requires the determination of $PSD(f_{x},f_{y})$. The definition of *PSD* is given by

(6) $$PSD(v_{x},\nu_{y})=\frac{\Delta x\Delta y{\left|S(v_{x},\nu_{y})\right|}^{2}}{N_{x}^{2}N_{y}^{2}}$$

where *N* represents the number of samples in the profile data, Δ denotes the sampling interval, and $S(\nu_{x},\nu_{y})$ is the two-dimensional Fourier transform of the surface profile p(x,y), that is:

(7) $$S(v_{x},\nu_{y})=F[p(x,y)]$$

**Figure 6:**
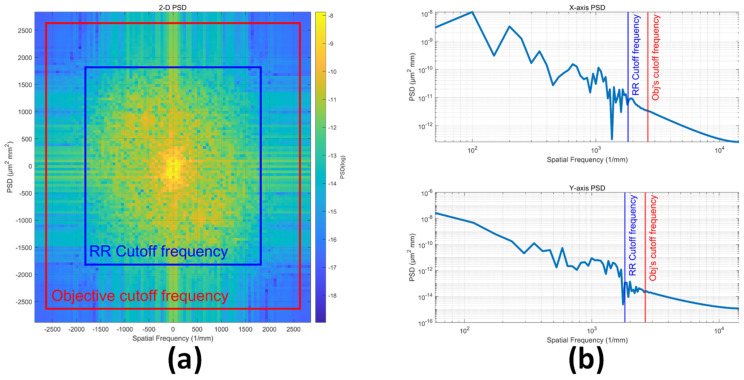
BRDF of the Cu pillar. (**a**) 2D PSD for the Cu pillar. (**b**) Cross−sectional PSD in x and y directions.

It is worth noting that the PSD data, in practice, is an approximation limited by bandwidth [24]. This limitation arises because 2D profile data, generated from a white-light interferometer, consists of samples with a finite number and interval. By substituting the profile data in Figure 1 into Equations (6) and (7), the PSD of the Cu pillar is determined, as shown in Figure 6. The blue box represents the bandwidth required by Rayleigh–Rice scattering theory under first-order approximation [22], and the red box denotes the instrumental bandwidth of the white-light interferometer [24]. The bandwidth of the profilometer adequately meets the requirements of Rayleigh–Rice scattering theory, which is crucial for accurate BRDF calculations.

**Figure 7:**
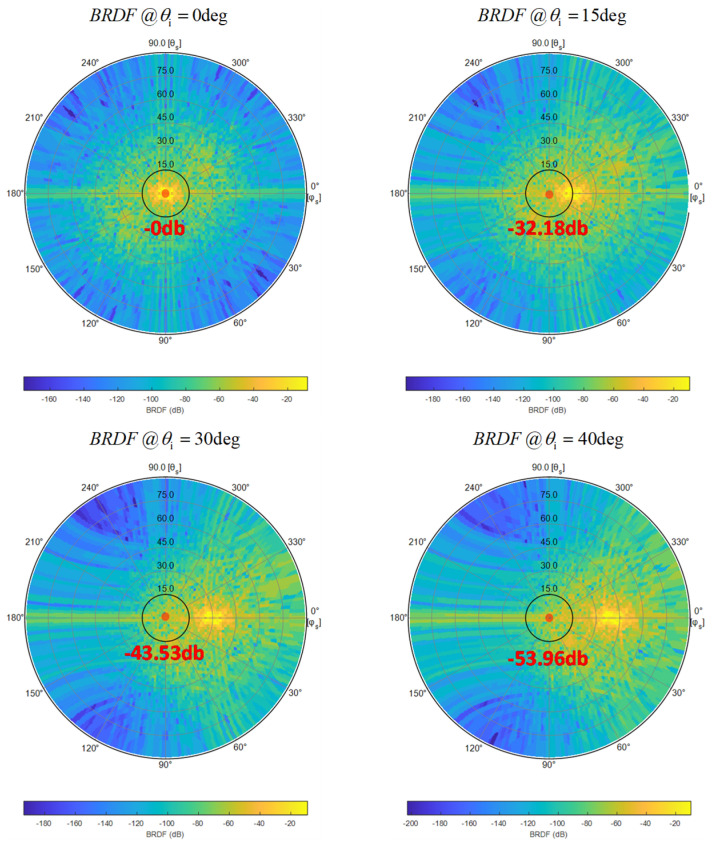
BRDF for the Cu pillar. The angles of incidence $\theta_{i}$ are 0°, 15°, 30°, and 40°.

The scattered light distribution of the Cu pillar is derived by substituting the PSD data from Figure 6 into Equation (2). Figure 7 illustrates the BRDF at various angles of incidence, annotating that scattered light (perpendicular to the top of the Cu pillar) attenuates relative to specularly reflected light.

#### 3.1.3. Detectable Region of Cu Pillar

**Figure 8:**
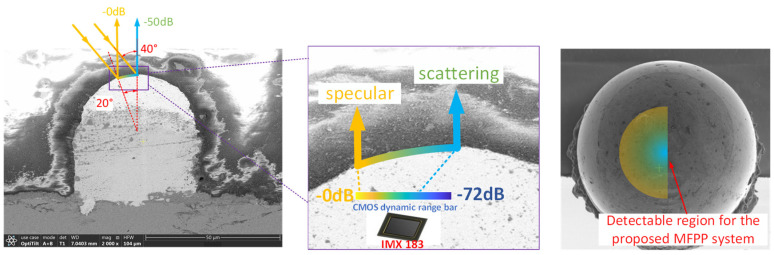
The detectable region corresponding to a specific CMOS sensor at a 40° angle of incidence of the DLP.

According to the BRDF, when the angle of incidence is 40°, the intensity of scattered light at the top of the Cu pillar attenuates by approximately −50 dB compared to the intensity of specularly reflected light. Due to the fact that CMOS typically has a dynamic range of 70 dB, it theoretically detects the scattered signal from the position corresponding to specular reflection up to the top of the Cu pillar, as shown in Figure 8.

In conclusion, we determined the BRDF of scattered light on the surface of the Cu pillar using Rayleigh–Rice scattering theory. The BRDF indicates that the CMOS (dynamic range is 72 dB) can detect scattered light on the top of the Cu pillar when the DLP’s angle of incidence does not exceed 40°. While the theoretically derived results show promise, the validity of the proposed MFPP system needs experimental verification.

### 3.2. Three-Dimensional Sensing Principle

#### 3.2.1. Phase Shifting Algorithm

The proposed MFPP system utilizes the 10-step least-squares phase-shifting algorithm (LS-PSA). To ensure sensing sensitivity, the fringe period is 10 DMD pixels for measuring profile, and the period is 40 DMD pixels for temporal phase unwrapping. At a structured light angle of incidence of 40°, the absolute phase corresponding to 40 DMD pixels spans approximately 220 μm vertically. Given that the expected height of the Cu pillar is 50 μm, this fringe period is reasonable.

Although the phase can be demodulated by projecting two sets of fringe patterns that satisfy the 10-step LS-PSA. However, in practice, to improve the measurement efficiency of the proposed MFPP system, the two sets of fringe patterns are composited into dual-frequency patterns using the temporal multiplexing principle [25], thereby avoiding the need to project additional patterns for phase unwrapping. Introducing a time-dependent phase factor, the wavefunction of the fringe patterns is given by

(8) $$\begin{array}{c} I\left(x,y,t\right)=a+b_{H}\cos\left(2\pi f_{H}x-M\omega_{0}t\right)+b_{L}\cos\left(2\pi f_{L}x-\omega_{0}t\right)\\ \omega_{o}=2\pi /10\\ t=\left\{0,1,2\ldots ,9\right\} \end{array}$$

where *x* and *y* are the number of pixels. When an 8-bit image is used, a=255/2, $b_{H}=0.8a$, $b_{L}=0.2a$. The spatial frequency $f_{H}=1/10$, $f_{H}=1/40$. The coefficient of frequency multiplication M=3.

The phase demodulation algorithm is given by

(9) $$\begin{array}{c} \begin{array}{c} \phi_{H}\left(x,y\right)=\tan^{-1}\left[\frac{\sum_{t=0}^{9}I\left(x,y,t\right)\sin\left(\frac{3\cdot 2\pi t}{10}\right)}{\sum_{t=0}^{9}I\left(x,y,t\right)\cos\left(\frac{3\cdot 2\pi t}{10}\right)}\right] \end{array}\\ \phi_{L}\left(x,y\right)=\tan^{-1}\left[\frac{\sum_{t=0}^{9}I\left(x,y,t\right)\sin\left(\frac{2\pi t}{10}\right)}{\sum_{t=0}^{9}I\left(x,y,t\right)\cos\left(\frac{2\pi t}{10}\right)}\right] \end{array}$$

Using the hierarchical algorithm [26], the absolute phase is computed, that is,

(10) $$\begin{array}{c} \Phi \left(x,y\right)=\phi_{H}\left(x,y\right)+2\pi \cdot k\left(x,y\right)\\ \begin{array}{c} k\left(x,y\right)=\mathrm{Round}\left[\frac{\left(f_{H}/f_{L}\right)\phi_{L}\left(x,y\right)-\phi_{H}\left(x,y\right)}{2\pi }\right] \end{array} \end{array}$$

where Φ(x,y) is the absolute phase and k(x,y) is the fringe order for each pixel.

#### 3.2.2. Three-Dimensional Point Cloud Registration

In the MFPP system proposed, both the projection and imaging modules utilize telecentric optics. The maximum vertical measurement range is sufficiently small relative to the telecentric lens’s depth of field, ensuring a linear relationship between height and phase. In this study, the reference-plane-based 3D reconstruction method [26] is used. It involves subtracting the absolute phase maps of the reference and measurement planes pixel by pixel and then multiplying by the phase-height coefficients $C_{p-h}$ to generate the height map:

(11) $$h(x,y)=C_{p-h}\cdot [\Phi_{\mathrm{Sur}}(x,y)-\Phi_{\mathrm{Ref}}(x,y)]$$

**Figure 9:**
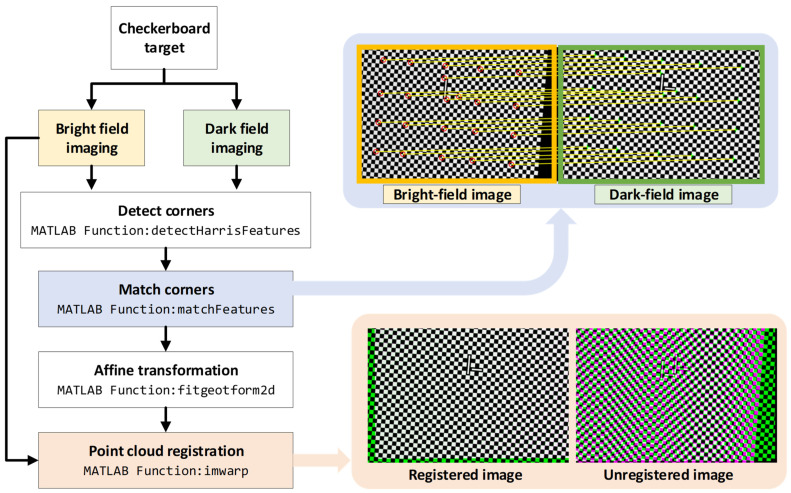
Flowchart of point cloud registration and intermediate results.

The height maps from the bright-field and dark-field imaging channels need registration for Cu pillar height evaluation. The telecentric optical system’s telecentricity and low aberration allow the use of a checkerboard target for point cloud registration. In this study, the control point-based image registration method [27] is used. Figure 9 illustrates the registration process using MATLAB(2023b)’s built-in library functions.

## 4. Experiment

**Figure 10:**
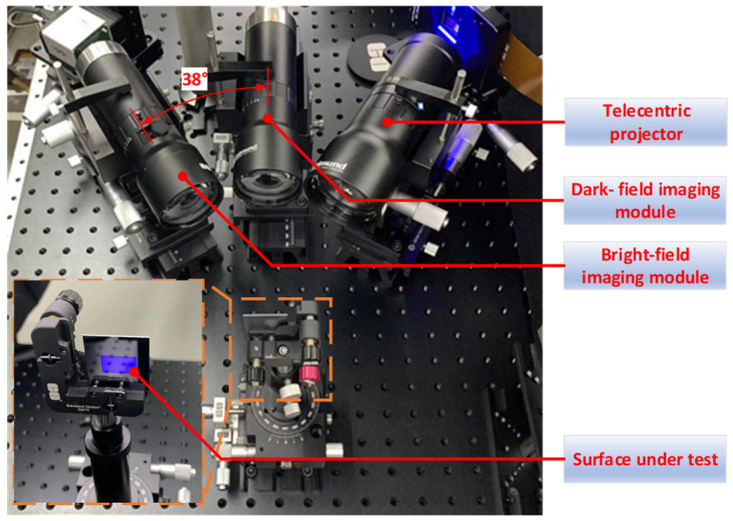
Experimental MFPP system.

Figure 10 shows the experimental setup for the proposed MFPP concept. The projector comprises a digital micromirror device (DMD, Texas Instruments, Dallas, TX, USA, DLP670s) and a telecentric lens (Edmund, 62#902, Shanghai, China). The bright-field imaging module includes an industrial camera (IDS, U3-3800SE-M-GL, Obersulm, Germany) and a telecentric lens (Edmund, 15#873, Shanghai, China). The dark-field imaging module shares identical components with the bright-field module. The optical axes of the bright field view are inclined at a 38° angle relative to the normal direction of the test surface. The light source used was a low-coherence LED with a central wavelength of 465 nm. To mitigate higher-order harmonics from the discrete pixel of the DMD, the telecentric lens in the projector is set to a f-number of 12. Additionally, to maximize the effective FOV of the MFPP system, the DMD and the CMOS in the bright-field module meet the Scheimpflug condition. Further, the focal planes of the bright-field and dark-field modules are precisely aligned using a method enabling visualization of their position and orientation [28].

### 4.1. Validation of the Detectable Region for Cu Pillar

**Figure 11:**
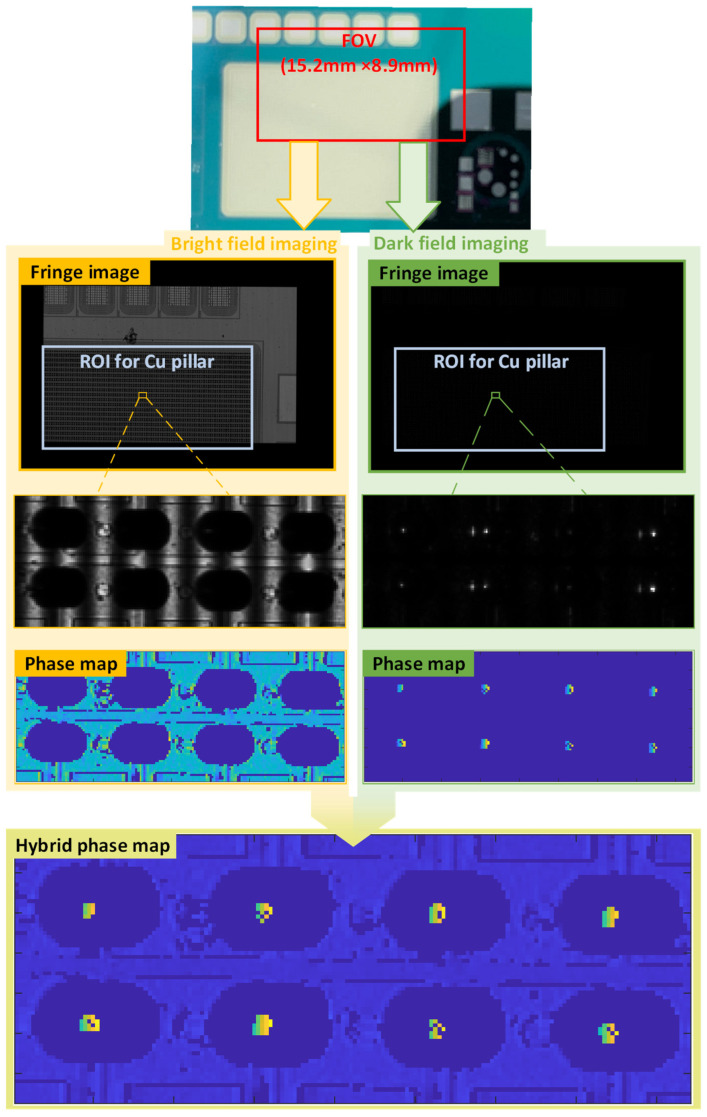
Intermediate results for phase demodulation.

**Figure 12:**
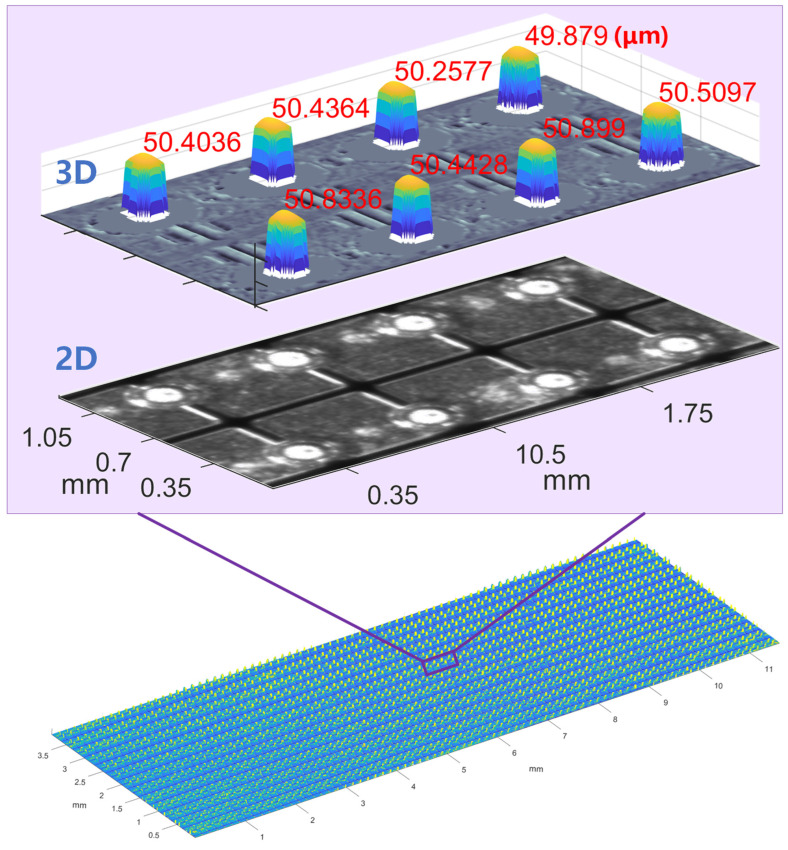
Three-dimensional reconstruction of wafer-1.

To verify the detectable region of the proposed MFPP system for Cu pillars, this experiment focuses on whether dark-field imaging can detect the top region of the Cu pillar. The surface under test was a sliced wafer containing Cu pillar structures with an expected height of 50 μm. Figure 11 shows intermediate results of the phase demodulation process for the bright field and dark field imaging modules. In practice, occlusion, weak scattering, and multiple reflections generate invalid point clouds. The modulation threshold is used as a mask to remove invalid point clouds from the phase map. Then, the bright field and dark field phase maps are fused. The phase of the top region of the Cu pillar can be measured from the fused phase map. Figure 12 shows the 3D reconstruction with the height of the Cu pillar labeled in the zoomed-in panel. Note that invalid point clouds outside the detectable region are bilinearly interpolated but do not affect the measured Cu pillar height values. Furthermore, no spatial smoothing was applied to the point clouds from either bright-field or dark-field imaging.

The experimental results demonstrate that the proposed MFPP system can detect the profiles of both the top of the Cu pillar and the substrate. In particular, the dark-field imaging module’s detection ability aligns with predictions based on the Rayleigh–Rice scattering theory shown in Figure 8. It is worth noting that Figure 11 shows a few isolated pixels in the dark-field phase map. These pixels are invalid point clouds caused by insufficient modulation and would appear as spikes without the modulation mask. In practice, the Cu pillar surface is not uniform but contains randomly distributed microstructures such as pits and bumps. These microstructures create surface defects that disturb the scattering distribution. This results in a localized scattered light intensity much lower than predicted by scattering theory. Consequently, insufficient scattered light cannot be responded to by CMOS, producing invalid point clouds. However, if the area occupied by these surface defects at the top of the Cu pillar is small, they theoretically do not significantly affect the height measurements. Conversely, if the Cu pillar is heavily scratched or corroded, detecting surface scattering signals becomes more difficult and unpredictable, requiring further investigation.

### 4.2. Measurement and Performance Evaluation

We evaluate the performance of the proposed MFPP, focusing on its maximum effective FOV and the measurement error in Cu pillar height, using another sliced wafer (wafer-2) as the test surface. The Cu pillar array on wafer-2 exceeds the MFPP system’s maximum FOV compared to wafer-1, facilitating evaluation of the system’s effective FOV. Additionally, the Cu pillar heights measured by the white-light interferometer served as the ground truth for evaluating measurement errors.

**Figure 13:**
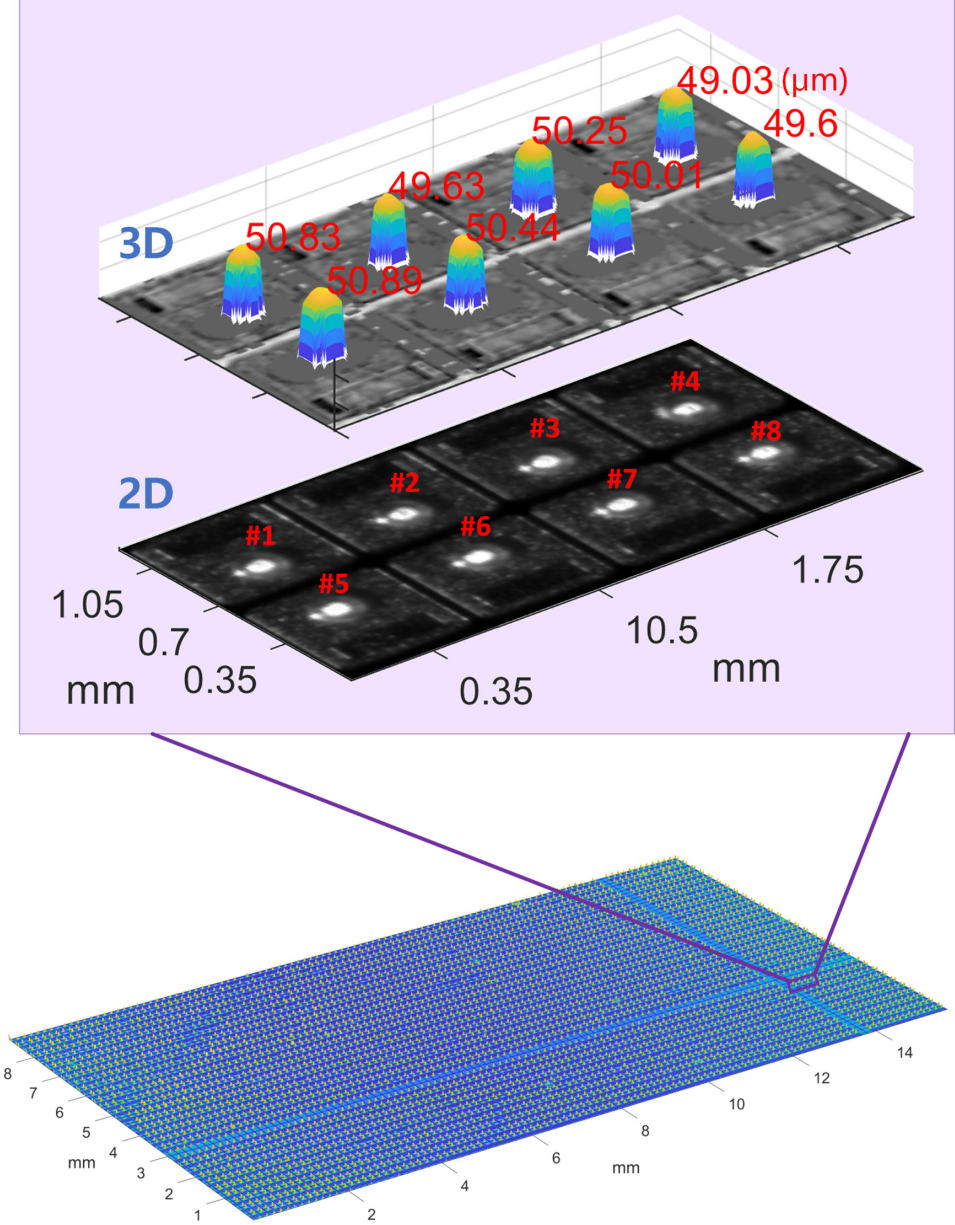
Three-dimensional reconstruction of wafer-2.

Figure 13 shows the 3D reconstruction of wafer-2. The reconstructed area covers 15.2 mm × 8.9 mm, defining the effective FOV of the proposed MFPP system. Note that the effective FOV is slightly smaller than the imaging module’s maximum FOV, as the DLP’s fringe pattern does not fully cover the FOV of the telecentric lens.

**Figure 14:**
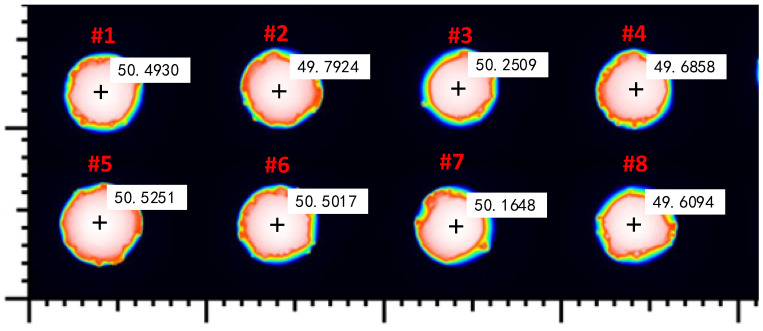
Cu pillar height, measured by a white-light interferometer.

**Figure 15:**
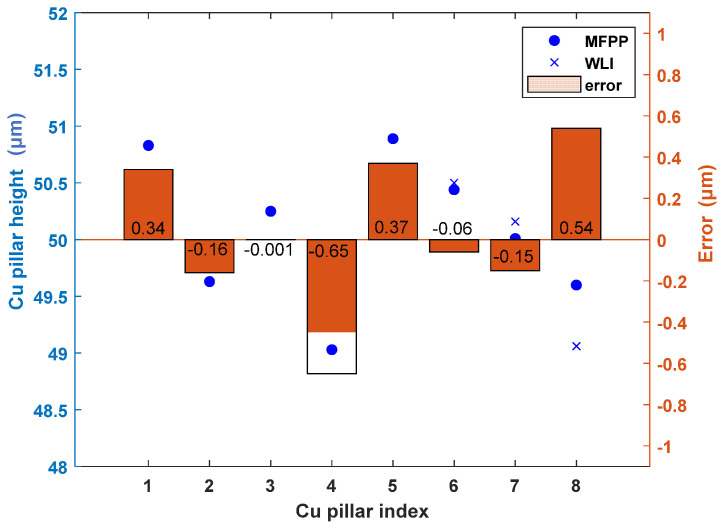
Measurement errors comparing the proposed MFPP system with a white−light interferometer.

To evaluate measurement errors, we used a white-light interferometer (SENSOFAR, Barcelona, Spain, S-neox, 20× objective) to measure the sub-panel region in Figure 13. Figure 14 shows the white-light interferometer measurements annotated with Cu pillar heights. Figure 15 shows the measurement errors for each Cu pillar. The maximum error is 0.65 μm, with an average error of 0.0286 μm. Measurement errors may stem from differences in spatial resolution between the proposed MFPP system and the white-light interferometer. Small scratches on Cu pillar surfaces may prevent the MFPP system, with its strong spatial filtering effect, from accurately measuring their heights. In fact, spatial resolutions vary among profilometers, resulting in deviations in profile measurement results. Further investigation is needed to figure out the impact of the proposed MFPP system’s spatial resolution on measurement results.

## 5. Conclusions

In conclusion, this study proposes a hybrid bright-dark-field microscopic fringe projection profilometry (MFPP) concept for measuring Cu pillars with large curvature and smooth surfaces in wafer-level packaging. Based on rigorous surface scattering theory, the MFPP system can detect optical signals from both the top of the Cu pillar and the substrate simultaneously, enabling efficient wide-field height measurement. Experimental results demonstrate that the effective field of view of the proposed MFPP system is 15.2 mm × 8.9 mm, and the MFPP system measured the height of the Cu pillar with a maximum error of 0.65 μm, which offers extremely wide-field bump height measurement compared to the white-light interferometer. The proposed MFPP concept has great application prospects in on-line bump height measurement in wafer-level packages, as well as effective wide-field 3D measurement for other micro-nano structural surfaces and devices, both in integrated circuits and MEMS. Additionally, the rigorous surface scattering theory applied to MFPP system design can also be adopted in the design of other systems aimed at measuring complex microstructures.

## Data Availability

Data are contained within the article.
